# Growth hormone: lessons from chickens

**DOI:** 10.3389/fphys.2025.1705885

**Published:** 2025-11-21

**Authors:** Colin G. Scanes

**Affiliations:** Department of Biological Sciences, University of Wisconsin Milwaukee, Milwaukee, WI, United States

**Keywords:** growth hormone, chicken, stress, lipolysis, growth

## Introduction

In chickens, the anterior pituitary gland produces the same palette of hormones seen across the vertebrates:

- Adrenocorticotropic hormone (ACTH) and β-endorphin
- Follicle-stimulating hormone (FSH)
- Growth hormone (GH)
- Luteinizing hormone (LH)
- Prolactin
- Thyrotropin (TSH)
- Neuropeptides, e.g.,

◦ Met-enkephalin
◦ Relaxin 3 (Lv et al., 2022)

This discussion focuses on contributions of the author and his collaborators with comments on what is still not known.

## Control of GH release and synthesis

Chicken somatotrophs respond to GH-releasing hormone (GHRH) and some neuropeptides. Intra-cellular concentrations of calcium ions in somatotrophs are increased by GHRH, thyrotropin-releasing hormone (TRH) (3/4 of somatotrophs), pituitary adenylate cyclase-activating peptide (85% of somatotrophs), leptin (51%), gonadotropin-releasing hormone (GnRH) (40%), and ghrelin (21%) (Scanes et al., 2007). Table 1 summarizes the neuropeptides that influence the release of GH (reviewed: Scanes, 2022). Some neuropeptides affect the release of more than one hormone. For instance, neuropeptide W decreases the secretion of GH, prolactin, and ACTH in chickens (Bu et al., 2016; Liu et al., 2022). What are still unknown are the following:

- Why there are multiple stimulatory and inhibitory factors?
- How pituitary cells influence the functioning of others?
- What the role of folliculostellate cells is?

These produce growth factors/hormones including annexin 1, fibroblast growth factor 2 (FGF2), leptin, and vascular endothelial growth factor (VEGF), and these presumably exert paracrine effects (Zhang et al., 2021).

**TABLE 1:** Summary of the hypothalamic releasing hormones/neuropeptides influencing the secretion of GH (based on discussions in Scanes, 2022).

Releasing hormone/neuropeptide	GH
GHRH	+
GnRH	+?
Ghrelin	+
Leptin	+
NPW	−
PACAP	+
SRIF	−
TRH	+

+ indicates increase; − indicates decrease.

## GH isoforms

There are multiple forms of GH in the chicken pituitary gland:

- Monomer (40%)
- Glycosylated (16%)
- Dimer (14%)
- 15–16 kDa sub-monomeric isoform (16%) (Luna et al., 2005)

The sub-monomeric isoform of GH predominates in immune tissues (Luna et al., 2005) and retinal ganglion cells in chickens (Baudet et al., 2003).

## GH and growth

The hypothalamo-pituitary GH–insulin-like growth factor-1 (IGF-1) axis exists in chickens and other birds. GH increases growth in hypophysectomized young chickens (King and Scanes, 1986). Growth is reduced in sex-linked dwarf chickens with a mutation(s) in the GH receptor gene (Burnside et al., 1991). Plasma concentrations of IGF-1 are reduced in hypophysectomized young chickens and restored by GH treatment (Huybrechts et al., 1985). GH increases IGF-1 release from chick hepatocytes (Houston and O’Neill, 1991) and in adult chickens (Radecki et al., 1997). The mechanism for GH’s effect on growth is mediated via Janus kinase (JAK)-2 (Zhou et al., 2005). Studies addressing the question as to whether GH increases growth in intact broilers are at best equivocal (Leung et al., 1986; Vasilatos-Younken et al., 1988; Cogburn et al., 1989; Scanes et al., 1990).

## GH and thyroid hormones

GH decreases hepatic deiodination of triiodothyronine (T_3_) in young chickens (Darras et al., 1992) with optimal circulating concentrations of T_3_ essential for growth. GH-receptor-deficient dwarf chickens have reduced plasma concentrations of T_3_ (Scanes et al., 1983).

## GH and lipolysis

Mammalian and avian GH stimulates *in vitro* lipolysis (glycerol release from adipose tissue explants) (Campbell and Scanes, 1985) and inhibits glucagon-induced lipolysis (Campbell and Scanes, 1987). A GH antagonist prevents GH’s effect on lipolysis *per se* but unexpectedly retains full activity in suppressing glucagon-induced lipolysis (Campbell et al., 1993). Moreover, reptilian, amphibian, and fish GH lacks lipolytic activity but inhibits glucagon-induced lipolysis (Campbell et al., 1991). What are not known are the following:

- Are the effects of GH physiologically relevant?
- Are these direct effects on adipocytes, or are these effects mediated through other cell types present in adipose tissue, such as endothelial cells and macrophages, followed by paracrine effects of cytokines or other neuropeptides?

The lipolytic effect is probably mediated through JAK-2 based on studies in mice (Shi et al., 2014). However, the mechanism for anti-lipolytic effects is yet to be determined.

## GH and reproduction

Administration of GH to laying hens increases shell thickness (Donoghue et al., 1990); this may be due to effects on the oviduct. This observation was followed by reports of oviductal and ovarian effects of GH. For instance, GH increases progesterone release from large yellow follicles (Hrabia et al., 2014a). GH decreases mucosal apoptosis in the oviduct but increases the expression of a specific gene (Hrabia et al., 2014b; Socha et al., 2017). Moreover, GH is present in the testes and ovary of chickens (Luna et al., 2014). There are associations between GH polymorphisms and egg production (Su et al., 2014).

## Stress and GH

Stress affects GH levels in post-hatch chickens. Plasma concentrations of GH were depressed following challenge with ACTH (Davison et al., 1980). Plasma concentrations of GH are also decreased by epinephrine (Harvey and Scanes, 1978) and morphine (Harvey and Scanes, 1987). Heat stress did not affect plasma concentrations of GH in young chickens but depressed hepatic expression of the GHR (Uyanga et al., 2022). Corticosterone induces somatotrophs in chick embryos (e.g., Bossis and Porter, 2000). Plasma concentrations of GH are increased by nutritional deprivation such as withholding feed or feeding a protein-deficient diet (Buonomo et al., 1982); the latter being presumed to be due to dietary stress depressing negative feedback for T_3_ and IGF-1.

## GH and the brain

Both GH- and prolactin-containing neurons are present within avian brains (Ramesh et al., 2000). Chick embryo retinal ganglion cells express GH (reviewed: Harvey et al., 2003; Harvey et al., 2012). Moreover, GH exerts a neuroprotective role in reducing apoptosis of retinal ganglion cells (Sanders et al., 2005). *In vitro*, GH depresses apoptosis and expression of caspase-3 and apoptosis-inducing factor-1 in neural retina explants from chick embryos (Harvey et al., 2006). Apoptosis in retinal ganglion cells is increased by antisera to GH *in ovo*, supporting a role for locally produced GH in regulating retinal apoptosis (Sanders et al., 2005). In an avian model for ischemic stroke, GH exerts a neuroprotective effect on cultured chick embryo hippocampal cells exposed to oxygen–glucose deprivation (Olivares-Hernández et al., 2021). Moreover, GH influences neurite development in the inner ear with increases in extension and branching of neurites in chick embryos (Gabrielpillai et al., 2018). Information on the underlying mechanism(s) for neuronal effects of GH is lacking.

## GH and angiogenesis

Chick embryo chorioallantoic membranes (CAMs) are useful for examining the effects of hormones and growth factors on angiogenesis. Clapp et al. (1993) reported that “*the 16-kilodalton N-terminal fragment of human prolactin is a potent inhibitor of angiogenesis*” using chick embryo CAMs. In contrast, the formation of blood vessels was stimulated by either an anterior pituitary tissue or GH (Gould et al., 1995). The signal transduction mechanism for these effects remains unclear.
